# A Review of Proteins Associated With Neuroprotection and Regeneration in Alzheimer's Disease

**DOI:** 10.7759/cureus.30412

**Published:** 2022-10-18

**Authors:** Harshita Agrawal, Ashok M Mehendale

**Affiliations:** 1 Psychiatry, Jawaharlal Nehru Medical College, Datta Meghe Institute of Medical Sciences, Wardha, IND; 2 Preventive Medicine, Jawaharlal Nehru Medical College, Datta Meghe Institute of Medical Sciences, Wardha, IND

**Keywords:** alzheimer's disease, neuronal degeneration, proteins, dementia, neuro-regenerative proteins, alzheimer's

## Abstract

One of the most prevailing conditions of dementia is the illness known as Alzheimer's disease. The diagnostic signs of Alzheimer's disease progressively get worse over a long period since it is a cumulative condition. Alzheimer's disease causes modest memory loss in its initial stages, but people cannot converse or react to their surroundings in the later stages of the disease. In Alzheimer’s disease, the destruction of neurons and the interconnection between them in the cortical region and the hippocampus is the beginning, after which the disease proceeds. The cerebral cortex regions are subsequently involved in thinking, linguistics, and interpersonal communication. Other parts of the brain eventually suffer harm as well. A person with Alzheimer's slowly loses the capacity to live and do daily tasks on their own over time. The illness is lethal in the end. Dementia is most commonly caused by ageing. Although dementia grows more prevalent as individuals age, this does not imply that dementia is a natural component of ageing. Up to 40% of those over 85 years who have dementia suffer from this condition. Amyloid, a beta protein that wrongly builds up and creates neurofibrillary tangles in the brain, causes Alzheimer's, a condition of protein misfolding. According to tradition, the primary cause of neuronal degeneration caused by the amyloid hypothesis is the buildup of beta-amyloid peptides. According to theory, the hazardous protein form that upsets the cell's calcium ion balance clumps amyloid fibrils, which leads to apoptotic cell death. This review article discusses the pathophysiology and biochemistry of various neuroprotective proteins to examine the potential of future anti-medications for Alzheimer's disease.

## Introduction and background

Intracellular neurofibrillary tangles and extracellular plaques containing tau and amyloid are indicatives of Alzheimer's disease (AD), a neurodegenerative condition. Amnestic cognitive impairment is the most frequent form of AD manifestation, while non-amnestic cognitive impairment may rarely appear [1]. Estimates indicate that 6.2 million Americans aged 65 years and over suffer from Alzheimer's dementia. If no medical advances are made to prevent or cure AD, there may be 13.8 million AD sufferers worldwide by 2060 [2]. In contrast to reported deaths from AD, which increased by more than 145% between 2000 and 2019, deaths from HIV, heart disease, and stroke all decreased between 2000 and 2019 [2]. AD is characterized by five stages: preclinical AD, AD with mild cognitive impairment (MCI), AD with mild dementia, AD with moderate dementia, and AD with severe dementia [2].

Preclinical Alzheimer's disease

Patients have measurable brain changes that are the disease's early biomarkers but have not yet manifested symptoms like memory loss. When the early symptoms (such as forgetting about recent conversations or events, misplacing items, and having trouble thinking of the right word) of preclinical AD appear, the brain makes up for them, allowing people to carry on with their daily lives as usual. It is essential to keep in mind that not everyone who exhibits signs of AD-related brain changes eventually develops MCI or dementia as a result of AD [2].​​​​​​

Mild cognitive impairment due to Alzheimer's disease

AD-related MCI patients show biomarker evidence of altered brain structure in addition to subtle memory and cognitive issues. These cognitive issues may be apparent to the person, family, and friends but not others, and they do not affect the person's capacity to carry out daily tasks. When the brain cannot repair the harm and death of nerve cells brought on by AD, mild cognitive changes occur. After two years, 15% of MCI patients start to experience dementia. Within a five-year follow-up period, 32% of MCI patients develop Alzheimer's dementia [2].

Dementia due to Alzheimer's disease

Alzheimer's dementia, also known as AD dementia, is distinguished by evident memory, thinking, or behavioural symptoms that impair a person's ability to function in daily life. People with advanced AD frequently experience multiple types of evolving symptoms. The extent of the harm done to the nerve cells in various brain areas is reflected in these symptoms. Each person experiences dementia differently, progressing from mild to moderate to severe symptoms at different rates [2].

Mild Alzheimer's dementia

Most people with mild Alzheimer's dementia are still able to function independently in many situations, but they may need assistance with some tasks to maintain their safety and level of independence. It is possible that they can continue to drive, work, and engage in their hobbies [2].

Moderate Alzheimer's dementia

The moderate stage of Alzheimer's dementia, which is frequently the most protracted stage, is characterized by changes in personality and behaviour, including suspicion and agitation, as well as communication and performance difficulties with daily activities (such as dressing and bathing) [2].

Severe Alzheimer's dementia

**Figure 1:**
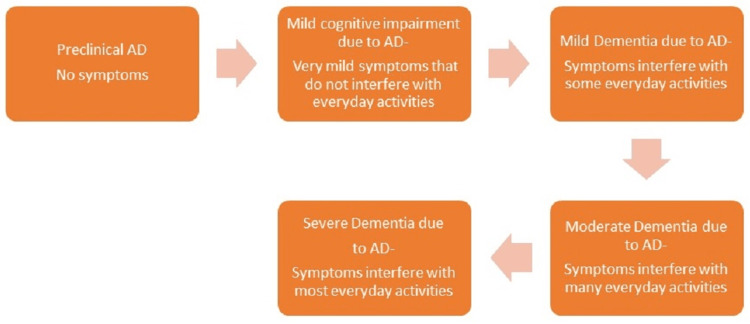
Stages of Alzheimer’s disease (AD) This image depicts the progression of Alzheimer's disease in an individual [2].

In the advanced stages of Alzheimer's dementia, patients need assistance with daily tasks and probably need round-the-clock care. At this stage, AD's effects on a person's physical health stand out, mainly because people become bedridden due to damage to the brain's movement-related regions. It is challenging to eat and drink when parts of the brain that regulate swallowing are damaged. Because of this, some people may swallow food into their trachea instead of their oesophagus. As a result, food particles may become lodged in the lungs, leading to an infection [2]. Various stages of AD are depicted in Figure 1 [2].

AD is a deceptive, complex illness that damages neurons and impairs cognitive function. Proteins are crucial in the pathogenesis of AD, along with altered genes and enzymes. Some proteins connected to AD include amyloid precursor protein (APP), glial fibrillary acidic protein, calmodulin-like skin protein, and matrix metalloproteinase-2. These proteins are essential to the AD hypothesis, which encompasses the damage to cholinergic neurons, the amyloid-beta (Aβ) hypothesis, and the tau hypothesis. The relevance of essential proteins and their physiological roles in the early diagnosis of AD is highlighted in the current review. Memory loss, synaptic malfunction, and cognitive decline are all caused by altered protein expression [3]. The significant neuropathological signs of AD seem to be neurofibrillary tangles and senile plaques. As the illness advances, the senile plaques start forming in brain regions connected to cognition before spreading to other cortical regions. The amyloid p-peptide (Aβ), a portion of the APP, is insoluble in senile plaques, among other things. Aβ peptide is produced from APP by two subsequent cleavage events: β-secretase produces one end of the peptide by proteolytic activity, and γ-secretase produces the other end through the same process [4].

Furthermore, even though specific other proteins might cause neurodegeneration, others, such as skin proteins with calmodulin-like properties, interact with heterotrimeric humanin receptor (htHNR) to stop it. Mutant presenilin-1 can increase the production of Aβ through the degradation of APP by presenilin-dependent secretase [5]. Pentraxin interactions are seen with all neurofibrillary tangles and senile plaques, in contrast to proteins like ubiquilin-1, which only interacts with APP and stimulates the production of insoluble Aβ peptides [6].

## Review

Methodology

In this study, we conducted focused literature searches using databases such as PubMed and Google Scholar to identify relevant original research and review articles. Search terms focused on Alzheimer's disease and associated proteins, and associations of neuro-protective and neuro-regenerative proteins in Alzheimer's disease. Titles and abstracts from these searches were reviewed, full-text articles were obtained for relevant manuscripts, and reference lists were reviewed to identify additional manuscripts appropriate for review. This is a traditional review and not a systemic review, so PRISMA (Preferred Reporting Items for Systematic Reviews and Meta-Analyses) or AMSTAR (Assessment of Multiple Systematic Reviews) guidelines were not followed.

Neuro-protective and neuro-regenerative proteins

Calmodulin-Like Skin Protein and Humanin

Calmodulin-like skin protein (CLSP), with 146 residues and a mass of 15.92 kDa, and calmodulin have 49% of the same amino acid sequence [7]. CLSP, a secreted peptide, interacts with the htHNR and starts an intracellular survival pathway to prevent neuronal cell death, which is connected to AD. Similar epithelial cells only found in skin keratinocytes expressing CLSP are capable of bridging the blood-cerebrospinal fluid (CSF) barrier [8]. Humanin inhibits a family gene associated with AD from causing neuronal cell death by interacting with the htHNR (AD). Humanin may thus perform a preventive function in the development of AD [9]. The differential characteristics of CLSP and humanin are mentioned in Table 1 given below [9]. Aβ is not necessary for CLSP-mediated neuroprotection or the improvement of memory impairment. The amounts of Aβ, soluble Aβ oligomers, or gliosis are not affected by CLSP. Additionally, CLSP prevents signal transducer and activator of transcription 3 (STAT3) inactivation and the disappearance of the synaptic marker synaptophysin in the hippocampus. The CLSP-1 gene, which produces CLSP, also lessens synaptophysin loss and the deficiency in spatial learning, and it activates the Janus kinase 2 (JAK2)/STAT3-mediated pro-survival signalling cascade in neurons via htHNR [10].

**Table 1:** Features of CLSP and humanin This table describes the differential characteristics of CLSP and humanin [9]. CLSP: calmodulin-like skin protein; CNS: central nervous system; BBB: blood-brain barrier.

	Humanin	CLSP
Amino acid length	24	146
Gene location	The exact location of the gene is unknown but is hypothesized to be in the mitochondria	Nucleus
Expression	Full expression profile unknown but assumed to be ubiquitous	Epithelial cells of the thyroid, mammary gland, prostate, kidney, and skin
Strongest expression	Testis	Skin
Entrance into CNS via BBB	Controversial	Yes

Heat Shock Protein

To defend themselves against stressful situations such as exposure to rapid heating, hypothermia, ultraviolet rays, wound repair, or tissue regeneration, cells create heat shock proteins (HSPs). The HSPs Hsp60, Hsp70, and Hsp90 have received the most investigation. HSPs are categorized according to their molecular weight [11,12]. The brain expresses HSPs as one of its many defences against oxidative stress. Patients with one or two copies of the HSP70-2 A2 allele in charge of the HSP70-2 protein's production exhibit noncognitive changes in AD [13].

HSP60, HSP70, and HSP90 expression levels in the brain's cerebellum were previously downregulated in AD. A critical transcription factor involved in the production of HSPs genes, heat shock factor 1 (HSF1), also undergoes a significant drop in the cerebellum. Nevertheless, increasing HSF1 expression aids in slowing Purkinje cell loss. In the brain's cerebellum, HSP60, HSP70, and HSP90 expression levels have previously been downregulated and considerably reduced AD [14].

Collapsin Response Mediator Protein 2

The family of proteins known as collapsin response mediators includes the phosphoprotein called collapsin response mediator protein 2 (CRMP-2). The amino acid sequence of CRMP-2, CRMP-1, CRMP-3, CRMP-4, and CRMP-5 are remarkably similar (50-70%), and their sizes vary from 60 to 66 kDa. Axon development from neurites is aided in the early stages by an excess of CRMP-2. Growth cone collapse results from CRMP-2 being phosphorylated by rho-kinase. CRMP-2 is phosphorylated by glycogen synthase, which also binds to and regulates microtubule formation, and this process is regulated [15].

Low-Density Lipoprotein Receptor-Related Protein 1

Low-density lipoprotein (LDL) receptor-related protein 1 regulates the brain parenchyma, neurons, astrocytes, microglia, vascular smooth muscle cells, and pericytes in the cerebrovasculature. Clearance at the blood-brain barrier is made more accessible by the ease of Aβ transfer from the brain to the circulation. These pathways regulate the Rho family GTPase activity to govern the RhoA-dependent endocytosis process, regulate the trafficking of other Aβ receptors like heparan sulphate proteoglycan (HSPG) and PrPc, and alter actin polymerization, and are probably involved in lipoprotein receptor-related protein 1 (LRP1)-mediated cellular Aβ uptake. In light of this, cellular Aβ clearance by LRP1 is probably handled through several endocytic routes depending on the type of brain cell [16]. LRP1 communicates with proteins implicated in the synthesis of Aβ, such as APP, beta-site amyloid precursor protein cleaving enzyme 1 (BACE1), and presenilin (PS), and its expression is reduced at the blood-brain barrier in AD patients. Additionally, it has been demonstrated that LRP1 regulates the impact of apoE on microglial inflammatory response in cell culture systems and mediates the relationship between apoE and cholesterol levels in the CNS via APP [17].

Mortalin

The capacity of mitochondrial proteins to function is maintained by the HSP mortalin (mtHsp70). Overexpression of mortalin exhibited the opposite effect, inhibiting mPTP activation and guarding SH-SY5Y neurons against Aβ-induced degeneration. Additionally, when exposed to Aβ, neurons overexpressing mortalin demonstrated improved control over the regulation of free calcium in the cytoplasm, a decline in the production of reactive oxygen species in the mitochondria, and a reduction in the Bax/Bcl-2 ratios in comparison to the control group [18]. Mortalin binds with a wide range of proteins, including APP, ApoE, fibroblast growth factor 1, HSP60, p53, 94 kDa glucose-regulated protein, and protein Dj-1, which in turn influences its range of activities, including cell survival, regulation of cell proliferation, and stress response [19].

It is found that overexpression of mortalin efficiently attenuated Aβ(1-42)-induced cell viability damage and apoptosis. Mortalin-specific small interfering RNA (siRNA) oligonucleotides significantly increased the susceptibility of SH-SY5Y cells to the neurotoxicity brought on by Aβ by inhibiting the expression of mortalin (1-42). Additionally, Aβ(1-42) reduced the activity of cytochrome c oxidase and adenosine 5′-triphosphate (ATP) production while increasing reactive oxygen radicals and lipid peroxidation. These effects were reversed entirely by overexpressing mortalin. However, the up-regulation of mortalin remarkably reduced Aβ-mediated mitochondrial fragmentation and cytotoxicity [20].

Neurogranin

Neurogranin, primarily generated in dendritic spines and involved in post-synaptic signalling pathways, regulates the calcium-binding protein (CBP) calmodulin. In model organisms and genetic studies, neurogranin has been linked to cognitive function and synaptic plasticity [21]. Neurogranin (Ng) is reportedly a key biomarker of injuries, but it also shows that neurons and dendrites are being repaired and regenerated [22]. According to Huang et al., Ng is thought to promote synaptic plasticity and long-term memory, and this concept was supported in their study. Its high concentration in neurons and propensity for binding to Ca2+-free CaM, which delays direct Ca2+ interaction with CaM by a "mass action" mechanism and effectively raises the free [Ca2+]i to enhance synaptic responses, are the most likely causes of this [23].

P-glycoprotein

Another element in the elimination of amyloid from the brain is P-glycoprotein. This 170 kDa plasma membrane protein is generated in humans by the multi-drug resistance of one gene and is a member of the ATP-binding cassette transporter family (MDR1 or ABCB1). The blood-brain barrier, the liver, the kidney, and the gastrointestinal tract are a few examples of the organs that express P-glycoprotein. On the luminal surface of epithelial cells at the blood-brain barrier, P-glycoprotein, an efflux transporter to release chemicals from the brain into the blood, is significantly expressed [24]. Increasing P-glycoprotein production has the potential to have two beneficial effects: accelerating the clearance of Aβ and reducing the pathologic levels of Aβ peptides [25]. On the other hand, its inhibition could accelerate the neurodegeneration linked to AD brought on by Aβ [26].

Ubiquitin

It is generally recognized that ubiquitination, a reversible post-translational modification of cellular proteins, is essential for controlling several cellular functions, including cell cycle regulation, protein degradation, DNA repair, and apoptosis [27]. The degree of neurofibrillary alterations in the tissue closely mirrored the increase in cerebral cortex ubiquitin levels in patients with Alzheimer's. The increase in ubiquitin levels in the cerebral white matter was noticeably less pronounced. The cerebellum's grey and white matter, unaffected by neurofibrillary changes in AD, had normal ubiquitin levels. The increase in ubiquitin reactivity was probably only partially mediated by the presence of ubiquitin in paired helical filaments (PHF). Tau accounts for the bulk of PHF, and only minimal amounts of ubiquitin have been explicitly related to PHF [28]. Normal neurons, plaque core amyloid, and tangle preparations soluble in sodium dodecyl sulfate (SDS) do not succeed in immunostaining. They also do not result in ubiquitin-positive blots [29].

Insulin and Insulin-Like Growth Factor-I

Reduced energy economy and low glucose absorption are two early symptoms of AD. These pathways are mediated by insulin and insulin-like growth factors (IGF) I and II. Serious problems are brought on by impaired insulin and IGF-mediated signalling and decreased energy utilization but, by activating the ERK-mediated pathway in astrocytes, insulin promotes the development of the enzymes that break down insulin and neprilysin, which are both necessary for the breakdown of Aβ [30]. Oligomers cause the breakdown of plasma membrane insulin receptors via a pathway sensitive to the inhibition of calcium- and casein-dependent kinase II (insulin receptor substrate (IRs)). Furthermore, insulin can stop this downregulation of surface IRs [31,32]. Coordinating hippocampal-dependent spatial learning and regulating astrocytic mitochondrial activity depends on IGF-I signalling. AD may be brought on by age-related astrocytic dysfunction brought on by decreased IGF-I signalling [33].

Calbindin-D28K, Calretinin, and Parvalbumin

Calbindin-D28K, calretinin, and parvalbumin are the three CBPs. Although calcium is essential for healthy brain development and operation, prolonged high levels of calcium in the brain can harm neurons and trigger cell death [34]. Early AD causes selective degeneration of the entorhinal cortex, which is connected to memory-related brain circuits. Non-principal neurons carrying calretinin, however, were better retained even in Alzheimer's patients with significant entorhinal disease, according to research by Mikkonen et al. [35]. Initially, in the AD entorhinal pathophysiology, non-principal cells harbouring parvalbumin or calbindin-D28k had morphological alterations. In AD, the vulnerability of neurons with various CBPs varies. Neurons expressing parvalbumin and calbindin-D28K start to deteriorate in layer II of the lateral, middle, and caudal subfields and in layer III in the most severe types of AD. Calretinin neurons of the entorhinal cortex exhibit less degradation in AD than parvalbumin-containing neurons [35]. These CBPs are indirectly linked in specific ways to the pathophysiology of AD, and by controlling intracellular calcium, the condition's course can be somewhat slowed [36].

## Conclusions

This review concentrates on the proteins connected to AD-related neuroprotection and regeneration. It is thought that mitochondrial cells are involved in the progression of AD because mitochondria are cytotoxic to an amyloid-beta peptide. A variety of proteins, including CLSP, humanin, HSP, CRMP-2, calbindin-D28K, calretinin, parvalbumin, LDL receptor-related protein 1, mortalin, and others, can have neuroprotective effects. It may be possible to lessen the neurotoxicity caused by Aβ by protecting mitochondria with these proteins. Research on proteins mentioned above in AD is urgently needed, as these might be promising targets for creating anti-AD treatments. In contrast, new potential biomarkers for diagnosing AD include C-reactive protein, pentraxins, CRMP-2, and growth-associated protein-43. In the future, a unique and effective pharmaceutical or disease-modifying approach that targets these proteins for the management and therapy of AD may become available.
